# A survey of simian *Plasmodium* infections in humans in West Kalimantan, Indonesia

**DOI:** 10.1038/s41598-022-21570-0

**Published:** 2022-11-03

**Authors:** Sri Riyati Sugiarto, Diana Natalia, Dayang Shuaisah Awang Mohamad, Nawal Rosli, Wendy A. Davis, J. Kevin Baird, Balbir Singh, Iqbal Elyazar, Paul C. S. Divis, Timothy M. E. Davis

**Affiliations:** 1 Medical School, Fremantle Hospital, University of Western Australia, PO Box 480, Fremantle, WA Australia; 2 Universiti Malaysia Sarawak (UNIMAS) Malaria Research Centre, Kota Samarahan, Sarawak Malaysia; 3 Faculty of Medicine, Universitas Tanjungpura, Pontianak, Indonesia; 4 Oxford University Clinical Research Unit (OUCRU) Indonesia, Jalan Diponegoro 69, Jakarta, 10430 Indonesia; 5 Centre for Tropical Medicine and Global Health, Nuffield Department of Medicine, University of Oxford, Oxford, UK

**Keywords:** Malaria, Epidemiology

## Abstract

The simian parasite *Plasmodium knowlesi* is the predominant species causing human malaria infection, including hospitalisations for severe disease and death, in Malaysian Borneo. By contrast, there have been only a few case reports of knowlesi malaria from Indonesian Borneo. This situation seems paradoxical since both regions share the same natural macaque hosts and *Anopheles* mosquito vectors, and therefore have a similar epidemiologically estimated risk of infection. To determine whether there is a true cross-border disparity in *P. knowlesi* prevalence, we conducted a community-based malaria screening study using PCR in Kapuas Hulu District, West Kalimantan. Blood samples were taken between April and September 2019 from 1000 people aged 6 months to 85 years attending health care facilities at 27 study sites within or close to jungle areas. There were 16 *Plasmodium* positive samples by PCR, five human malarias (two *Plasmodium vivax*, two *Plasmodium ovale* and one *Plasmodium malariae*) and 11 in which no species could be definitively identified. These data suggest that, if present, simian malarias including *P. knowlesi* are rare in the Kapuas Hulu District of West Kalimantan, Indonesian Borneo compared to geographically adjacent areas of Malaysian Borneo. The reason for this discrepancy, if confirmed in other epidemiologically similar regions of Indonesian Borneo, warrants further studies targeting possible cross-border differences in human activities in forested areas, together with more detailed surveys to complement the limited data relating to monkey hosts and *Anopheles* mosquito vectors in Indonesian Borneo.

## Introduction

One of the aims of the Global Technical Strategy for malaria 2016–2030 developed by the World Health Organisation (WHO) was for a reduction in malaria case incidence and mortality rate of at least 40% between 2015 and 2020^1^. Malaysia is one of the countries to have achieved this goal, with zero reported indigenous non-zoonotic malaria cases since 2018. However, zoonotic malaria due to *Plasmodium knowlesi* increased from 1600 cases to > 4000 between 2016 and 2018 before declining to 3213 and 2609 cases in 2019 and 2020, respectively^1^. Malaysia is the nation with the highest reported incidence of human *P. knowlesi* infections in South-east Asia^2,3^ and, in the Malaysian Borneo states of Sabah and Sarawak, the majority of malaria cases and most malaria-associated hospitalisations are caused by *P. knowlesi*^4–6^. The presence of this parasite in jungle areas harbouring its natural macaque monkey hosts and *Anopheles* mosquito vectors is a clear impediment to achieving malaria eradication once human malarias have been eliminated^7,8^.

**Figure 1:**
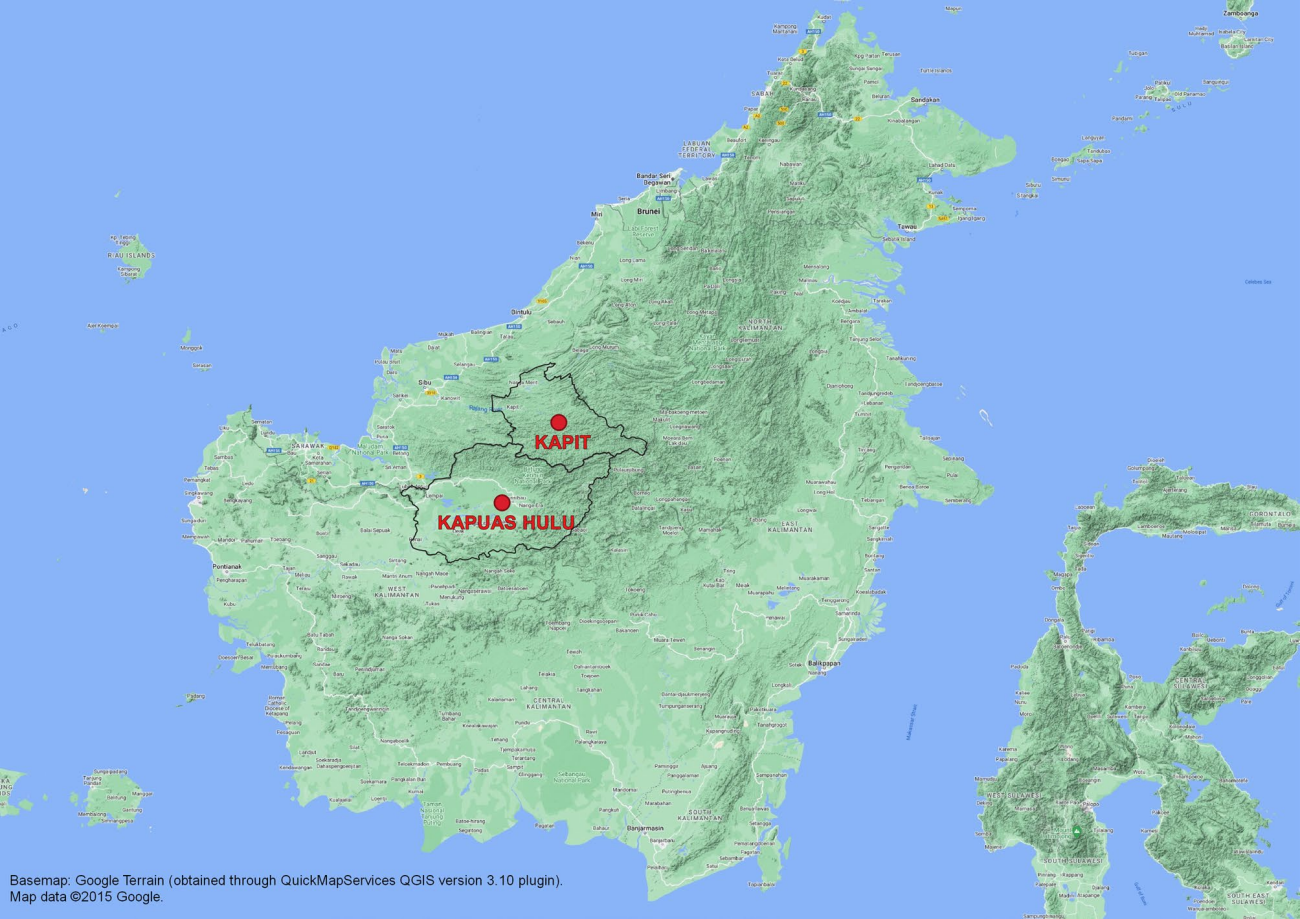
Map of the island of Borneo showing that the Kapit Division in Sarawak, Malaysia, is situated across the northern border of Kapuas Hulu District, West Kalimantan, Indonesia.

Indonesia is another South-east Asian country that has come a long way in controlling malaria. In the late 1940s, malaria was identified as a major public health problem with substantial implications for economic activity^9^, and 70 years later there were still large estimated numbers of clinical cases each year (1.3 million *Plasmodium falciparum* and 1.5 million *Plasmodium vivax* in 2010)^10,11^. However, enhanced control measures had reduced the burden of malaria to an estimated 0.3 million clinical cases of *P. falciparum* and 0.4 million clinical cases of *P. vivax* by 2021.^12^ The Indonesian government aims to eliminate malaria in stages by 2030, from the most to the least developed islands^13^. As in the example of Malaysia, transmission of *P. knowlesi* is likely to impede eradication efforts in Indonesia. Indeed, three Indonesian provinces (North, East and West Kalimantan) share a border with the Malaysian states of Sabah and Sarawak (see Fig. 1), and the natural hosts (*Macaca fascicularis* and *M. nemestrina*)^14^ and mosquito vectors (including *Anopheles leucosphyrus*)^14,15^ of *P. knowlesi* occur on both sides of the border. The predicted risk of knowlesi malaria is, therefore, similar for both Malaysian and Indonesian Borneo^16^.

Although there is a significant burden of knowlesi malaria in both Sabah and Sarawak, including severe and fatal cases^6,17–20^, data relating to the prevalence and clinical sequelae of this infection in humans in Indonesian Borneo are sparse. Only 12 confirmed cases have been reported to date^21–25^ and all of these are from South and Central Kalimantan rather than the provinces of West and North Kalimantan bordering Malaysian Borneo. Most data relating to *P. knowlesi* in Indonesia come from the approximately 400 cases found in Sumatra^26^ and Aceh^27^ provinces. The reasons for the disparity in the burden of *P. knowlesi* between Indonesian and Malaysian Borneo is unclear. Misdiagnosis may be contributory since the microscopic appearances of *P. knowlesi* can be interpreted as those of *Plasmodium malariae* or *P. falciparum*, and the more accurate but expensive nested PCR-based testing is rarely done outside research settings in Indonesia^19,28,29^. There could be greater awareness of knowlesi malaria as a diagnostic possibility in Malaysian Borneo than in Kalimantan given that it was first discovered as a significant public health problem around 20 years ago in the Kapit Division of Sarawak^30^. However, there may also be differences between Indonesian and Malaysian Borneo in demographic and environmental conditions impacting transmission.

To investigate the cross-border disparity in *P. knowlesi* prevalence, we conducted a community-based screening study using PCR in Kapuas Hulu District, West Kalimantan, the closest Indonesian district to the Kapit Division of Sarawak. We hypothesized that there would be previously unrecognised cases of *P. knowlesi* and perhaps cases of other simian malarias among people attending health care facilities and living and working in areas of the district in close proximity to forested areas.

## Results

**Figure 2:**
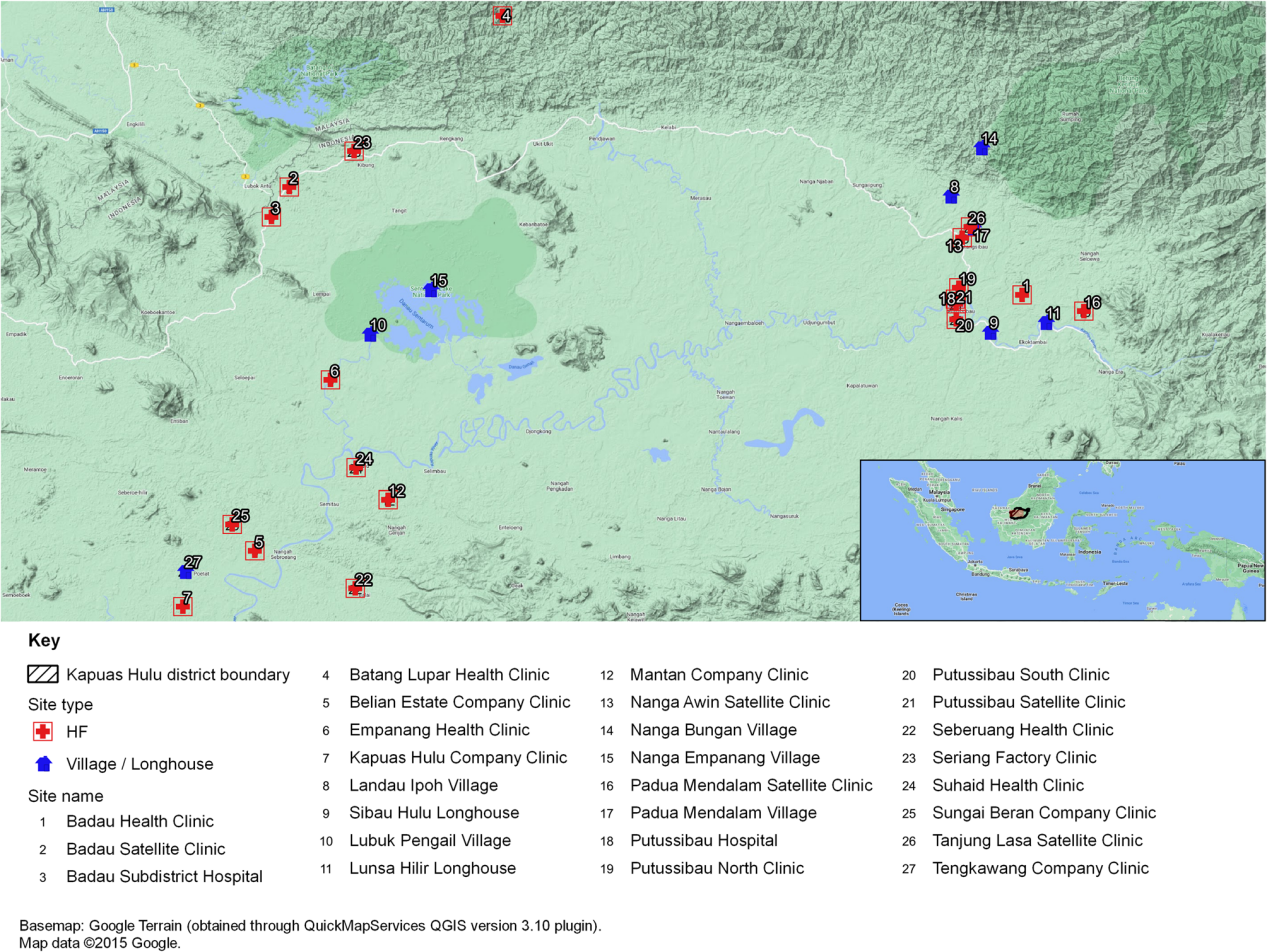
Health clinics and hospitals in Kapuas Hulu district where participants in the present study were screened for malaria.

A total of 1000 individuals were recruited from 27 sites in Kapuas Hulu District of West Kalimantan and, of these, 431 (43.1%) were from government health clinics (GHCs), 143 (14.3%) from palm oil plantation health clinics, 102 (10.2%) from satellite health clinics, 65 (6.5%) from villages, and 53 (5.3%) from longhouses (see Fig. 2 for locations and Supplementary Table 1 for full details of the individual sites selected and case distribution). The characteristics of the sample are summarised in Table 1. Participants ranged from 6 months to 85 years of age but the majority were adults of working age. The proportions of males and females were similar. Approximately one third were plantation workers and farmers from relatively high-risk malarious areas in the district. A third had fever or a recent history of fever. Compared to those who were afebrile, this sub-group was younger with proportionately more children and males. Although they were also less likely to be plantation workers and farmers, this may have reflected the larger numbers of people attending peripheral health care facilities for general health checks rather than because of symptoms.

**Table 1:** Sample characteristics.

	Total	Febrile	Afebrile	*P* value*
n (%)	1000	336 (33.6)	664 (66.4)	
Age (median [inter-quartile range])	37 [25–50]	30 [13–46]	39 [28–51]	
**Age group (n %)**				
< 1 year	4 (0.4)	4 (1.2)	0	0.013
1–5 years	55 (5.5)	40 (11.9)	15 (2.3)	< 0.001
6–12 years	60 (6.0)	39 (11.6)	21 (3.2)	< 0.001
13–29 years	224 (22.4)	73 (21.7)	151 (22.7)	0.748
30–50 years	423 (42.3)	114 (33.9)	309 (46.5)	< 0.001
> 50 years	236 (23.6)	66 (19.6)	168 (25.3)	0.048
**Sex (n %)**				
Male	499 (49.9)	179 (53.3)	319 (48.0)	0.124
Female	501 (50.1)	157 (46.7)	344 (51.8)	0.141
**Occupation (n %)**				
Home duties	199 (19.9)	58 (17.3)	141 (21.2)	0.154
Palm oil worker	181 (18.1)	14 (4.2)	167 (25.1)	< 0.001
Farmer	163 (16.3)	42 (12.5)	121 (18.2)	0.023
Infant/child	93 (9.3)	72 (21.4)	21 (3.2)	< 0.001
Casual worker	90 (9.0)	62 (18.4)	28 (4.2)	< 0.001
Student	76 (7.6)	28 (8.3)	48 (7.2)	0.530
Business owner	75 (7.5)	31 (9.2)	44 (6.6)	0.162
Retired	30 (3.0)	12 (3.6)	18 (2.7)	0.440
Fisherman	26 (2.6)	3 (0.9)	23 (3.5)	0.019
Public servant	24 (2.4)	5 (1.5)	19 (2.9)	0.273
Goldminer	16 (1.6)	1 (0.3)	15 (2.2)	0.017
Other	27 (2.7)	8 (2.3)	19 (2.9)	0.658
**Malaria diagnosis**				
Rapid diagnostic test	297 (29.7)	218 (64.9)	79 (11.9)	< 0.001
Microscopy	35 (3.5)	35 (10.4)	0	< 0.001
None	668 (66.8)	83 (24.7)	585 (88.1)	< 0.001

*For febrile versus afebrile participants by Chi-squared test.

There were four participants diagnosed with malaria at the recruitment site but with negative PCR (see Table 2), two with a positive RDT (one *P. falciparum* and one mixed *P. falciparum/P. vivax*) and the other two with a positive blood slide (one *P. falciparum* and one *P. vivax*). All were adults who presented with fever. Five cases, four of whom presented with fever and three of whom had an RDT which was negative, were positive by PCR and the infecting species was identified (two cases of *P. vivax* and two of *P. ovale*, and one case of *P. malariae*). Two of these cases were children and one an infant aged 6 months—all of these cases were febrile when recruited.

**Table 2:** Details of cases of malaria diagnosed by PCR with the species identified (cases 1–5), those who were PCR positive but without an identified *Plasmodium* species (cases 6–16), and those who were positive by rapid diagnostic test or microscopy but negative by PCR (cases 17–20).

No.	Age (years)	Sex	Occupation	Residence	Recruitment site	Fever	Rapid diagnostic test	Microscopy	PCR result
1	0.5	Female	Infant	Nanga Awin	Putussibau North Clinic	Yes	Not done	Not done	*P. malariae*
2	1	Male	Child	Mentebah	Putussibau Hospital	Yes	Negative	Not done	*P. vivax*
3	11	Male	Child	Lanjak	Batang Lupar Health Clinic	Yes	Negative	Not done	*P. ovale*
4	48	Male	Village head	Meriau	Batang Lupar Health Clinic	Yes	Negative	Not done	*P. vivax*
5	50	Male	Business owner	Nanga Kantuk	Putussibau Satellite Clinic	No	Not done	Not done	*P. ovale*
6	22	Male	Casual worker	Bunut Hulu	Putussibau Hospital	Yes	Not done	Not done	Species not identified
7	26	Male	Palm oil worker	Seriang	Badau Health Clinic	No	Not done	Not done	Species not identified
8	31	Female	Home duties	Kedamin Hulu	Putussibau South Clinic	No	Not done	Not done	Species not identified
9	34	Female	Fisherman	Nanga Empanang	Nanga Empanang village	No	Not done	Not done	Species not identified
10	36	Female	Farmer	Lanjak	Batang Lupar Health Clinic	Yes	Negative	Not done	Species not identified
11	38	Male	Palm oil worker	Belian Estate	Belian Estate Company Clinic	No	Not done	Not done	Species not identified
12	49	Male	Business owner	Kedamin Hilir	Putussibau Satellite Clinic	Yes	Negative	Not done	Species not identified
13	50	Female	Home duties	Nanga Lauk	Badau Health Clinic	Yes	Not done	Not done	Species not identified
14	62	Male	Public servant	Datah Dian	Putussibau Hospital	Yes	Not done	Not done	Species not identified
15	71	Female	Home duties	Lanjak	Batang Lupar Health Clinic	Yes	Negative	Not done	Species not identified
16	73	Male	Farmer	Silat Hulu	Putussibau North Clinic	No	Not done	Not done	Species not identified
17	24	Male	Casual worker	Putussibau	Putussibau North Clinic	Yes	*P. falciparum*	Not done	Negative
18	17	Female	Student	Badau	Badau Sub-district Hospital	Yes	Not done	*P. falciparum*	Negative
19	32	Male	Palm oil worker	Sui Tembaga	Badau Health Clinic	Yes	*P. falciparum/vivax*	Not done	Negative
20	29	Male	Soldier	Putussibau	Putussibau South Clinic	Yes	Not done	*P. vivax*	Negative

**Figure 3:**
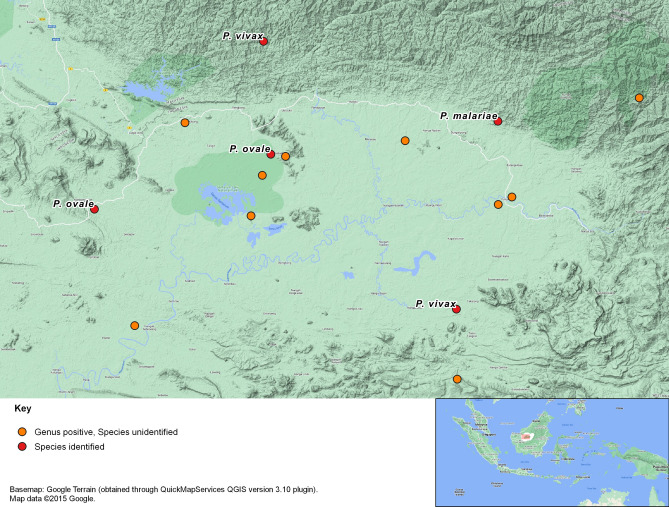
Map showing the locations at which the cases of PCR positive malaria were identified.

There were 11 cases, six of whom had fever, who were *Plasmodium*-positive by PCR but the species could not be identified by PCR assays with primers specific for *P. falciparum. P. vivax, P. malariae, P. ovale, P. knowlesi, P. cynomolgi, P. inui, P. fieldi* and *P. coatneyi.* We further characterized these 11 samples by amplifying the partial small subunit ribosomal RNA (SSU rRNA) genes and generating 233–234 base pair (bp) and 272–294 bp amplicons with two different pairs of Nest-2 PCR primers for direct sequencing. Analysis of the DNA sequences, after the PCR primer sequences were removed, was based on the Basic Local Alignment Search Tool (BLAST) from the National Center for Biotechnology Information (NCBI) database revealed the presence of *Plasmodium* species*,* with high sequence similarity to human and simian *Plasmodium* species (*P. knowlesi, P. inui, P. fieldi* and *P. coatneyi*) for seven of the 11 cases (see Table 3). However, the identity of the infecting *Plasmodium* species could not be inferred through phylogenetic analyses since the DNA sequences generated were relatively short, resulting in phylogenetic trees with low bootstrap values (see Supplementary Fig. 1). None of the 11 cases were diagnosed and treated for malaria at the time of recruitment since RDTs and microscopy were not done or were negative. Of the 16 PCR positive cases, there were four who presented at the Batang Lupar Health Clinic in the north of the survey area and another 11 at sites around Putussibau Hospital in the east, both areas close to the border with Sarawak, with only a single case in the west at the Belian Estate Company Clinic (see Fig. 3).

**Table 3:** Sequencing results for 11 *Plasmodium* genus positive samples based on the closest reference of *Plasmodium* species according to the BLAST search analysis of the SSU rRNA gene sequences. Samples from four cases (numbers 6, 12, 13 and 14 in Table 2) were not sequenced because the sequence length was < 100 bp. Samples from cases 7, 10 and 11 were sequenced using different primers or different Nest-1 products, or both.

No.	Primers	DNA sequence length (bp)	Closest *Plasmodium* species reference	Similarity
7	rPLU3 + rPLU4	183	P. inui clone B0362A91	100%
7	PlasmoM_N2F + PlasmoM_N2R	248	*P. inui* clone B0362A91	100%
8	rPLU3 + rPLU4	183	*P. knowlesi* isolate KM12k	100%
9	rPLU3 + rPLU4	183	*P. knowlesi* isolate KH96A-type	100%
10A	rPLU3 + rPLU4	174	*P. knowlesi* clone B0362B12	100%
10B	rPLU3 + rPLU4	174	*P. fieldi* clone LT3-B11	100%
11A	rPLU3 + rPLU4	183	*P. inui* clone B0362A91	100%
11B	rPLU3 + rPLU4	183	*P. inui* clone B0362A91	100%
11	PlasmoM_N2F + PlasmoM_N2R	226	*P. knowlesi* strain H	97.74%
15	rPLU3 + rPLU4	174	*P. knowlesi* clone B21-87	100%
16	rPLU3 + rPLU4	183	*P. coatneyi* isolate B136co	100%

## Discussion

The present data collected over a six-month period show that, in contrast to the epidemiological situation in neighbouring Kapit Division of Sarawak, Malaysian Borneo^4–6^, there were very few cases of malaria in Kapuas Hulu District, West Kalimantan, identified through screening people resident and/or working in theoretically high-risk areas, a third of whom had fever. In addition, there was no definitive evidence of *P. knowlesi* infection or other simian malarias in our samples. We found PCR evidence of human malarias except *P. falciparum* but, in most of the PCR-positive participants, the species of *Plasmodium* could not be accurately identified. This could indicate that there is low level parasitaemia of species such as *P. knowlesi* in Kapuas Hulu District, but affecting only up to approximately 1% of a population living in relatively close proximity to the natural hosts and mosquito vectors of *P. knowlesi*^16^.

**Figure 4:**
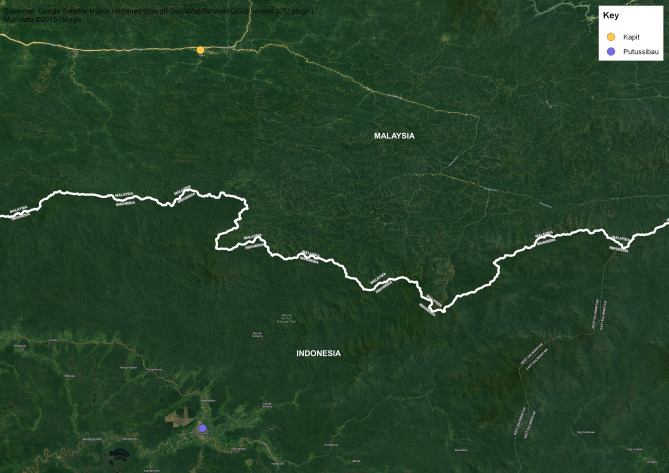
Sattelite view of Kalimantan-Sarawak border showing extensive road system on Sarawak side and a paucity of jungle access roads on the West Kalimantan side.

One possible difference between Indonesian and Malaysian Borneo in the risk of transmission of *P. knowlesi* is in ease of access to forested areas. On the Indonesian side, there are few roads penetrating densely vegetated areas compared to the more extensive road network on the Malaysian side (see Fig. 4). This may mean that forest-related occupations such as logging, mining and farming associated with potentially greater vector exposure are more common in Sarawak and Sabah than West Kalimantan. There may also be other ecological and demographic factors at play such as cross-border differences in deforestation, habitat fragmentation, agricultural expansion, socioeconomic changes and wildlife reservoirs that can influence *P. knowlesi* transmission^31^. It is not possible to determine whether the lack of zoonotic infections in the Kapuas Hulu District is due to smaller populations of the two macaque hosts of zoonotic malaria parasites compared with the geographically adjacent Kapit Division of Sarawak, Malaysian Borneo, since no data are available on macaque populations in these two regions, but the mosquito vectors are present on both sides of the border^29^. Greater economic development in Malaysian compared with Indonesian Borneo^32,33^ could result in an increased risk of zoonoses^34^. Some relevant Malaysian studies have been conducted^35–37^, including one in the state of Sabah in Malaysian Borneo examining potential associations between environmental variables obtained from satellite-based remote-sensing data and *P. knowlesi* incidence which showed that deforestation in areas surrounding villages was positively associated with numbers of *P. knowlesi* cases at a village level^38^. There is a relative paucity of such data from Kalimantan.

Our failure to identify the species of *Plasmodium* in a number of participants who were positive for *Plasmodium* by PCR has been observed in other surveys of malaria parasites in humans, non-human primates and mosquitoes^39–41^. One explanation for this is that relatively small volumes of blood with very low density infections may yield sufficient DNA for the *Plasmodium*-specific PCR assay targeting both the A- and S-types of the SSU rRNA genes, but not for the species-specific primers directed at only one type of gene^41^. Another possible reason is that these individuals were infected with a *Plasmodium* species other than the eight targeted by the species-specific primers, such as *P. inui-like* or *P. simiovale* which have recently been found to cause zoonotic infections^42,43^. Although BLAST searches were suggestive, phylogenetic analyses of the sequencing data were not definitive for species confirmation. It is, therefore, possible that a proportion of these individuals had sub-microscopic *P. knowlesi* infections that were asymptomatic or not the cause of fever, but the corollary to this is that there should be other people in the same areas with clinically evident knowlesi malaria requiring treatment in local healthcare facilities^27,39^ with some progressing to severe disease^6,17–20^. Even if *P. knowlesi* cases were misidentified by microscopy as one of the human malarias^19,28,29^, historical data showing low number of total malaria cases with relatively few hospitalisations in Kapuas Hulu District^12^ would suggest that the burden of knowlesi malaria in the community, if present at all, is small. By contrast, in the neighbouring region of Kapit Division in Sarawak, Malaysian Borneo, 83 knowlesi malaria patients were admitted to Kapit Hospital during the period during which the present survey was conducted, with 1246 and 973 annual cases of knowlesi malaria reported in 2018 and 2019, respectively, for the state of Sarawak, Malaysian Borneo (unpublished data, Sarawak State Health Department).

There were four cases of malaria (0.4%) diagnosed by microscopy or RDT who were negative by subsequent PCR, and three of the participants (0.3%) were PCR positive with a species identified but who were negative by RDT. False positive and negative microscopy results are well recognised in areas of low and unstable transmission such as Kapuas Hulu District^44^, and can occur even in the context of clinical trials^45^. False positive RDTs can result from persistence of antigen well after successful treatment^46^ or rarely in patients with cross-reacting antibodies^47^, while false negatives are usually due to parasitaemias below the limit of detection of the test^48^. The overall numbers of such cases in the present study were, therefore, consistent with other studies.

In conclusion, the results of the present survey are consistent with the very limited number of cases of knowlesi malaria reported to date from Indonesian Borneo^21–25^ which do not include any from the Kapuas Hulu District of West Kalimantan where the survey was conducted. Although we cannot rule out low-level and largely asymptomatic simian malarias including that due to *P. knowlesi*, evidence from Malaysian Borneo^39,40^ and other parts of Indonesia^27^ suggests that this would be expected to parallel symptomatic cases requiring treatment which are not observed in Kapuas Hulu District. There are likely to be significant differences in sociodemographic, economic and environmental factors that underlie the substantial disparity between the burden of knowlesi malaria either side of the Malaysian-Indonesian border on the island of Borneo. Nevertheless, there is a need to survey other areas along the Kalimantan-Sarawak and Kalimantan-Sabah border to determine whether the low prevalence of malaria, including simian *Plasmodium* infections, is confirmed.

## Methods

### Study design, site and approvals

The present study was a cross-sectional survey of malaria prevalence including speciation in people from Kapuas Hulu District in West Kalimantan Province. This district has a total area of 29,842 km^2^ and a population of 253,740 in the latest census by Indonesian Bureau of Statistics^49^. It was selected as the study site based on its close proximity to the Kapit division in Sarawak, Malaysia^30^ with which it shares its northern border (see Fig. 1). Kapuas Hulu has stable low malaria transmission with an Annual Parasite Incidence (API; number of positive cases per 1000 individuals in a year) of 0.13^12^. It has tropical climate with an average temperature of 26.7 °C and average annual precipitation of 4231 mm. The district comprises mainly forested and plantation areas. The population lives in towns, in villages by rivers or lakes, and in housing inside plantations.

Two district hospitals and 23 sub-district GHCs serve the district. For the purposes of the present study, screening was performed at both district hospitals (Achmad Diponegoro and Badau) and at seven of the GHCs (Putussibau Utara, Putussibau Selatan, Lanjak, Badau, Suhaid, Seberuang and Empanang; see Fig. 2). The sub-district GHCs in the present survey were selected based on ease of access and relatively high malaria prevalence from the previous year’s numbers reported to the Kapuas Hulu Health Department. To capture potentially at-risk individuals in remote areas who may not have been able to access available health care facilities, community-based screening was also performed in six palm oil plantation health clinics, five satellite health clinics (Pos Binaan Terpadu, *Posbindu*), five villages and two longhouses (see Fig. 2). The study was approved by Ethical Committee for Health Research of Diponegoro University, Indonesia (Ref. No. 608/EC/FK-UNDIP/X/2018) and Human Ethics of The University of Western Australia, Australia (Ref. RA/4/20/4879). The study was performed in accordance with relevant guidelines and regulations. Permits for screening were obtained from palm oil plantation companies and outreach services provided by health clinics.

### Sample size

Sample size calculations were based on the assumption that screening in hospital clinics, GHCs and more remote sites would increase the likelihood of identifying *P. knowlesi* in both symptomatic and asymptomatic individuals compared to that in the general Kapuas Hulu population. In a study from Sabah, a study of an enriched sample of household and community members of clinical cases showed a prevalence of 1.7% by PCR using the *P. knowlesi*-specific primers^39^. A sample size of ≥ 1000 would be sufficient to identify a prevalence of 1.0% greater than this with 1% precision and 95% confidence, with fewer numbers of participants required if the true prevalence were less^50^, as has been shown in some previous community-based surveys in Sabah^51^ and Sarawak^40^.

### Sample collection

Screening was conducted between April and September 2019. Between April and June, staff at the government health facilities were requested to take a dried blood spot (DBS) on filter paper, in addition to malaria blood film and/or or Rapid Diagnostic Test (RDT), from all patients presenting with fever. Because the numbers of participants recruited in this way was low, and given emerging evidence that asymptomatic cases of knowlesi malaria would be missed^40,51^, screening between July and September 2019 was extended to all people utilizing outpatient services at the various health facilities provided that they (or their parent/guardian in the case of children) gave witnessed informed consent to sampling and had been resident in the area for at least 6 months. In addition to a DBS for PCR, basic demographic details and a history of symptoms were taken, and testing for malaria by microscopy (single read as negative or positive with speciation but without quantification or semi-quantitative ‘plus’ system grading of parasite density) and/or RDT (CareStart™, Access Bio, Somerset, NJ, USA) was performed depending on the capacity of the local facilities. The same requirements and procedures were followed for community screening in which sample collection took place at the same time as outreach services to villages and longhouses by government health clinics. The outreach service was a routine monthly activity by nurses or health workers for villages with limited access to health care facilities. The individual sites and numbers of samples taken are summarised in Table 1.

Malaria DBS samples were collected from individuals with or without fever who had been resident in the area for a minimum of six months. A total of 1000 blood samples were obtained by finger prick using a spring-loaded lancet which were spotted onto filter papers (Whatman® Schleicher and Schuell® qualitative filter paper) for subsequent molecular analysis. Three separate blood spots per filter paper were collected from the same sampling site for each participant. The spotted filter paper was air dried and stored in an individual sealed plastic bag at room temperature until DNA extraction in the laboratory. Participants diagnosed with malaria by microscopy or RDT were treated according to national guidelines.

### DNA extraction and detection of Plasmodium DNA

DNA was extracted from DBS by using Instagene® Matrix (BioRad Laboratories, https://www.bio-rad.com) as described previously^52^. Nested PCR assays based on the *Plasmodium* SSU rRNA genes were used to screen DNA extracted from DBS^53^. Screening for presence of malaria parasites using *Plasmodium*-genus specific primers for the first amplification Nest-1 (rPLU1 and 5) and Nest-2 amplification (rPLU3 and 4) was first performed on all samples to generate an approximately 1.6 kb amplicon and a 240 base pair amplicon, respectively. For Nest-1, each sample was tested using reaction mixture of 50 μL consisting 0.25 μM of each primer (rPLU1 and rPLU5), PCR buffer (50 mM KCl, 10 mM Tris–HCL), 3 mM MgCl_2_, 200 mM each deoxynucleotide triphosphate, 1.25 U of Taq DNA polymerase (Promega, USA, https://www.promega.com) and 15 μL of DNA template. PCR amplification was performed using a ProFlex Thermal Cycler (Thermo Fisher, https://www.thermofisher.com) under the following conditions: 94 °C for 4 min, 35 cycles of 94 °C for 30 s, 55 °C for 1 min and 72 °C for 1 min, followed by 72 °C for 4 min. Two microlitres of the Nest-1 products were used as DNA template in the 20μL Nest-2 *Plasmodium* genus-specific assays under the following conditions: 94 °C for 4 min, 40 cycles of 94 °C for 30 s, 62 °C for 1 min and 72 °C for 30 s, followed by 72 °C for 4 min. Nest-2 PCR products were analysed by gel electrophoresis and SYBR® Safe (Invitrogen, USA) gel staining. For the *Plasmodium*-positive samples, the Nest-1 amplification products were reused for species-specific Nest-2 amplifications. All genus positive samples were analysed at least twice by two independent laboratory staff. Positive species-specific samples were confirmed by retesting and samples with inconsistent results were re-extracted before being re-analysed.

### Molecular detection of human and simian Plasmodium species

*Plasmodium*-positive samples were further tested for the identification of human and simian *Plasmodium* species using the species-specific PCR primers for *P. falciparum*, *P. vivax*, *P. malariae*, *P. ovale*, *P. knowlesi*, *P. inui*, *P. cynomolgi*, *P. fieldi*, and *P. coatneyi* in Nest 2 PCR assays as described previously^54^. The primers were rFAL1 and rFAL2 for *P. falciparum*, rVIV6 and rVIV7 for *P. vivax*, rMAL1 and rMAL2 for *P. malariae*, rOVA1 and rOVA4 for *P. ovale*, Kn1f. and Kn3r for *P. knowlesi*, PinF2 and INAR3 for *P. inui*, CY2F and CY4R for *P. cynomolgi*, PctF1 and PctR1 for *P. coatneyi* and PfldF1 and PfldR2 for *P. fieldi*^54^. Nest-2 PCR amplification was carried out in a 20μL reaction mixture containing identical amount of species-specific primers and other constituents apart from the addition of 2 mM MgCl_2_ and 0.5 U Taq DNA polymerase. Nest-2 PCR amplification conditions were akin to the of Nest-2 *Plasmodium*-genus PCR assay, except for the annealing temperature of 58 °C for three human *Plasmodium* species-specific primer pairs (*P. falciparum, P. ovale* and *P. malariae*)^55^, 65 °C for *P. vivax*, 62 °C for *P. knowlesi* and *P.coatneyi*, 60 °C for *P. cynomolgi* and *P. inui,* and 66 °C for *P. fieldi*. Similarly, the species-specific Nest-2 products were subjected to gel electrophoresis and were stained using SYBR Safe™. Precautions to prevent cross-contamination in nested PCR assays were taken as described previously^30^.

### DNA sequencing and analyses

PCR amplification of partial SSU rRNA genes was conducted using the *Plasmodium*-specific primers rPLU3 and rPLU4 as described above. In addition, we also amplified the partial SSU rRNA gene of approximately 252 bp long by independent hemi-nested PCR assays using primers PlasmoM_N1F and PlasmoM_N1R in the first PCR amplification, followed by primers PlasmoM_N2F and PlasmoM_N1R in the second PCR amplification as described previously^56^. Both amplified products were sent to Apical Scientific Sdn. Bhd. (Selangor, Malaysia) for direct DNA sequencing. The DNA sequences generated were confirmed for *Plasmodium* DNA sequence using BLAST from the NCBI website. DNA sequences were then aligned using the MegAlign software (DNAStar Lasergene, Madison, USA) together with other reference sequences of primate *Plasmodium* species (see Supplementary Fig. 1), and the Neighbour-Joining trees were generated using the MEGA X software^57^ with a bootstrap of 1000 replications.

## Supplementary Information

Supplementary Information.

## Data Availability

The datasets used and/or analysed during the current study are available from the corresponding author on reasonable request. The DNA datasets generated and/or analysed are available in the GenBank repository (Accession numbers OP020384 to OP020394).
