# Supplementary material for: A survey of simian Plasmodium infections in humans in West Kalimantan, Indonesia
Source: Sci Rep. 2022 Nov 3;12:18546. doi: 10.1038/s41598-022-21570-0 (PMC9633791; doi:10.1038/s41598-022-21570-0)
Supplement: Supplementary file 1 — Supplementary Information. [file 41598_2022_21570_MOESM1_ESM.docx]

**Supplementary Table 1.** Description of study sites and number of samples collected at each.

| Site | Characteristics | Number of DBS collected |
| --- | --- | --- |
| Putussibau Hospital | A type C referral hospital with 4 medical specialists, 10 general practitioners and 2 dentists. It has outpatient and inpatient facilities with 8 wards (paediatric, maternal, surgery and general). The laboratory does examinations for both inpatients and outpatients and may receive around 30-50 samples per day, but unfortunately it was understaffed and overworked. In this laboratory, blood spots were collected in only a small number by laboratory persons. This hospital is located in the town centre of Putussibau and easily accessible. Malaria screening is initially done by RDT (Carestart™) and if positive, thin blood slide will be made to identify the species. Only the microscopy result was recorded in the hospital record. | 124 |
| Badau Subdistrict Hospital | A type D hospital with visiting-only specialists and 4 general practitioners. It also has outpatient and inpatient facilities with 4 wards. The laboratory has a smaller number of patients and samples to handle, therefore the laboratory persons were able to make blood spots properly, yet staff were not able to make malaria slides. The hospital is located out of the town of Badau (at the border with Sarawak) and accessible by a dirt road. | 82 |
| Putussibau North Clinic | Easily accessed from Putussibau town, the clinic has a relatively well-equipped laboratory with reliable laboratory staffs. It also has 4 doctors, 1 dentist and a 24-hours emergency maternal delivery room for pregnant women. It covers 19 villages in the northern area of Putussibau. It is located in the same area as the district hospital. | 116 |
| Putussibau South Clinic | Conveniently accessible from Putussibau town by car. The laboratory has adequate facilities yet staff were not able to collect blood spots. It also has 4 GPs (all in temporary contracts), 1 dentist but only an outpatient clinic which runs during office hours. It covers 16 villages, some are very remote in upstream Kapuas River, in the southern area of Putussibau. | 133 |
| Batang Lupar Health Clinic | Situated in the north Kalimantan road route between Putussibau and Badau, Batang Lupar clinic has 2 doctors and a very modest inpatient facility. The clinic services and area which includes 10 villages mostly around Lake Sentarum National Park. The laboratory is one of the better resourced outside Putussibau town. Batang Lupar is the only subdistrict area that still reports malaria cases every year, although in the last 4 years the numbers have fallen substantially. | 79 |
| Badau Health Clinic | Located in the town centre of Badau, the northern town of Kapuas Hulu bordering Malaysia, Badau health clinic has both outpatient and inpatient facilities with a 24-hour Emergency Department. It has 3 doctors but no dentist. The laboratory is, unfortunately, not manned. Lab examinations were done by clinic staff using mainly rapid/dipstick tests. Badau Health Clinic provides basic medical services and public health services to 9 villages which mostly comprise palm oil plantation settlements. | 61 |
| Empanang Health Clinic | West of Badau town, out from the tarmac road is Empanang subdistrict. Empanang Health Clinic has 1 doctor and a basic medical service covering 6 villages in more forested areas. The laboratory staff comprises a trainee who was able to collect blood spots. Access is challenging via a muddy road and river, therefore visitors normally have to stay overnight. | 22 |
| Seberuang Health Clinic | Located on the southern road route from Putussibau, in the direction to Sintang district and Pontianak city, Seberuang is one of the oldest health clinic first built by Christian missionaries. It covers 15 villages (but not as densely populated as Putussibau town) and has 1 doctor. It provides outpatient service only. The laboratory was not manned either. | 19 |
| Suhait Health Clinic | Also in the southern region of Kapuas Hulu district, western side of Lake Sentarum, Suhait Health Clinic covers 11 villages. The villages are very diverse, from forested areas to palm oil plantation to stilted and floating house on lake and rivers. It has no doctor and no laboratory person, unfortunately, therefore only a few blood spots were collected only during the visit to the area. | 3 |
| Putussibau Satellite Clinic | It is the only satellite clinic that is located in town. This satellite clinic has a very important role for patients in Putussibau area as it gives service outside the office hours. It has a simple laboratory service and a doctor who is available in the evening. No inpatient facility with all emergency cases referred to the hospital. All samples that were taken from this clinic were from patients with fever who had mostly come from villages around Putussibau. Malaria screening is by microscopy. No malaria drugs are available; patients need to get medications from government clinics during office hours. | 37 |
| Padua Mendalam Satelite Clinic | This satellite clinic is located at Padua Mendalam village, a remote area under Putussibau North Clinic. Mostly consists of clustered houses around the river, the Punan Dayak village is dotted with churches as the main religion of the village is Catholic. The clinic’s main role is to deliver health programmes for babies and toddlers such as immunisation, growth and development, and nutrition. It also promotes programmes for the elderly and pregnant women. This clinic screens for HIV, glucose, uric acid and cholesterol for pregnant women but does not have RDT to screen for malaria. Their activities (*posyandu, posbindu*) take place in general once per month. It is accessible on partly tarmac and partly dirt road in an hour on motorbike from Putussibau town. | 9 |
| Tanjung Lasa Satellite Clinic | The satellite clinic is located at Tanjung Lasa village. Accessible by dirt road, this clinic has 1 midwife in residence to run the government health programmes for children, pregnant women and the elderly. No malaria screening or malaria drugs are available. | 12 |
| Nanga Awin Satelite Clinic | This satellite clinic has better access to Putussibau town (tarmac road) but the area is mostly forested areas along the river. The satellite clinic in this area serves specifically for pregnant women and children health programmes. No malaria screening or malaria drugs are available. | 21 |
| Badau Satellite Clinic | Easily accessible from Badau town, this area is closer to the Lubuk Antu (Malaysia) border. It is mostly inhabited by palm oil plantation casual workers, as Badau town is surrounded by palm oil plantation. The clinic is under Badau Government Health Clinic and also functions as a point of delivery for health programs such as promoting elderly and infant health from village to village, glucose and cholesterol screening, deworming for children, etc. No malaria screening or malaria drugs are available, but there is easy access to the town clinic via a tarmac road. | 23 |
| Kapuas Hulu Company Clinic | Located in the southern part of Kapuas Hulu district, it is owned by SinarMas company. The areas is around 5,000 ha and the number of workers is about 3,000 (including the casual workers). There is a clinic managed by a senior nurse and 2 nurse assistants. No regular malaria screening in place. Malaria drugs are available (chloroquine and sulfadoxine-pyrimethamine). | 20 |
| Tengkawang Company Clinic | Also located in the southern part of Kapuas Hulu district. The areas is around 8,400 ha and the number of workers is about 3,750 (including the casual workers). The company clinic is run by a medically-trained nurse and 1 assistant. Also no regular malaria screening is in place. Malaria drug availability is limited to chloroquine. | 18 |
| Belian Estate Company Clinic | One of the largest estate in the Southern part of Kapuas Hulu. The area is around 17,000 ha and the number of workers is about 5,800 (including casual workers). Their clinic is one of the most well equipped inside a palm oil company, with quite a large supply of drugs. No regular malaria screening but the company pays for indoor residual spraying and bednets. Malaria drugs available are dihydroartemisinin-piperaquine and Fansidar. Malaria drugs are not supposed to be given without a confirmed diagnosis, yet the distance to town discourages patients getting a diagnosis from the government health clinic. The company clinic is run by 1 trained nurse, 1 midwife and 3 health assistants. | 34 |
| Sungai Beran Company Clinic | Situated next to Belian estate, this clinic was also part of the palm oil company. It is run by two trained health workers (*mantri*) with visiting midwife from Belian estate. The area is around 6,500 ha and the number of workers is about 3,500 (including casual workers). | 20 |
| Mantan Company Clinic | The most remote palm oil plantation area, with low socioeconomic status and a large number of local workers. Located across the river in Southern area of Kapuas Hulu district (near Semitau hospital) with access this clinic via river oat. The area is around 2,800 ha and the number of workers is about 2,000 (including casual workers). The company clinic is managed by a midwife and 1 assistant. No malaria screening is available. Malaria drug availability is limited to chloroquine. | 19 |
| Seriang Factory Clinic | Located in the northern part of Kapuas Hulu district, near Badau area. Accessible only by a dirt road. This palm oil company is close to Sarawak border and the workers sometimes work in both countries. The area is around 15,000 ha and the number of workers is about 6,000 (including casual workers). The clinic is managed by a midwife and 2 trained assistants. There is no regular malaria screening and no malaria drugs are available as the Badau Government Health Clinic is accessible where people can get tests and medications. | 32 |
| Nanga Bungan village | This remote village is located upstream of Kapuas river, 1-day journey for one way and an overnight stay is necessary. Mostly inhabited by Dayak Kayan and the population work as gold miners (by manually filtering river sands for nuggets). This village is visited by Putussibau South Clinic once in every 6 months. | 16 |
| Lubuk Pengail village | This village is located in the Lake Sentarum area near Nanga Empanang. Most inhabitants work as fishermen. All houses are either on stilts or floating. The closest health facility access is Suhaid Health Centre. | 3 |
| Nanga Empanang village | This village is also located in the Lake Sentarum, in the east. Only accessible by boat and all houses are floating. People work as fishermen or merchants, bringing goods from the land to the lake. The village is visited once per 3 months by the Suhaid Health Clinic. | 17 |
| Landau Ipoh village | The village is accessible by river only, one hour using a long boat. Most houses are on stilts and there is no satellite clinic in the village, only a private practice midwife. The closest health clinic is Putussibau North Clinic. | 13 |
| Padua Mendalam village | A village outside Putussibau town area, accessible by dirt road. House to house visits are done to improve tuberculosis diagnosis, check mosquito breeding and bednets (relatively few) and malaria screening. | 14 |
| Lunsa Hilir Long House | It is an approximately 800 metres longhouse belongs to Iban Dayak from North of Putussibau area. It is accessible by land, by 4WD. There are more than 150 people living in this one long house, mostly working as farmers in the forested areas. | 52 |
| Sibau Hulu Long House | Stretching about 500 metres in length and housing around 100 people, this closest longhouse to South Putussibau is located at the forest fringe. The one patient living in this house had fever and chills - therefore a health worker was called to provide a malaria test and collect a blood sample. | 1 |

**Supplementary Figure 1:** Neighbour-Joining phylogenetic trees of *Plasmodium*-genus positive samples isolated from Kapuas Hulu, West Kalimantan, Indonesia based on the small subunit ribosomal RNA genes. Nucleotide sequences from the isolates are in bold, and were generated using (A) primers rPLU3 and rPLU4 with ~240 bp in length, and (B) primers Plasmo1 and Plasmo2 with ~252 bp in length. The bootstrap values at nodes were generated by 1,000 replicates, and only values above 70% are shown. In this result, the identity of the infecting *Plasmodium* species (samples in bold) could not be inferred through phylogenetic analyses since the DNA sequences generated were relatively short, resulting in phylogenetic trees with low bootstrap values. Samples KI 353 and KI 353 are from Participant no. 7, KI 809 is from Participant no. 8, KI 59 is from Participant no. 9, KI 334A and KI 334B are from Participant no. 10, KI 175A, KI 175B and KI 175 are from Participant no. 11, KI 978 is from Participant no. 15, and KI 676 is from Participant no. 16.

| **A** | **B** |
| --- | --- |
| 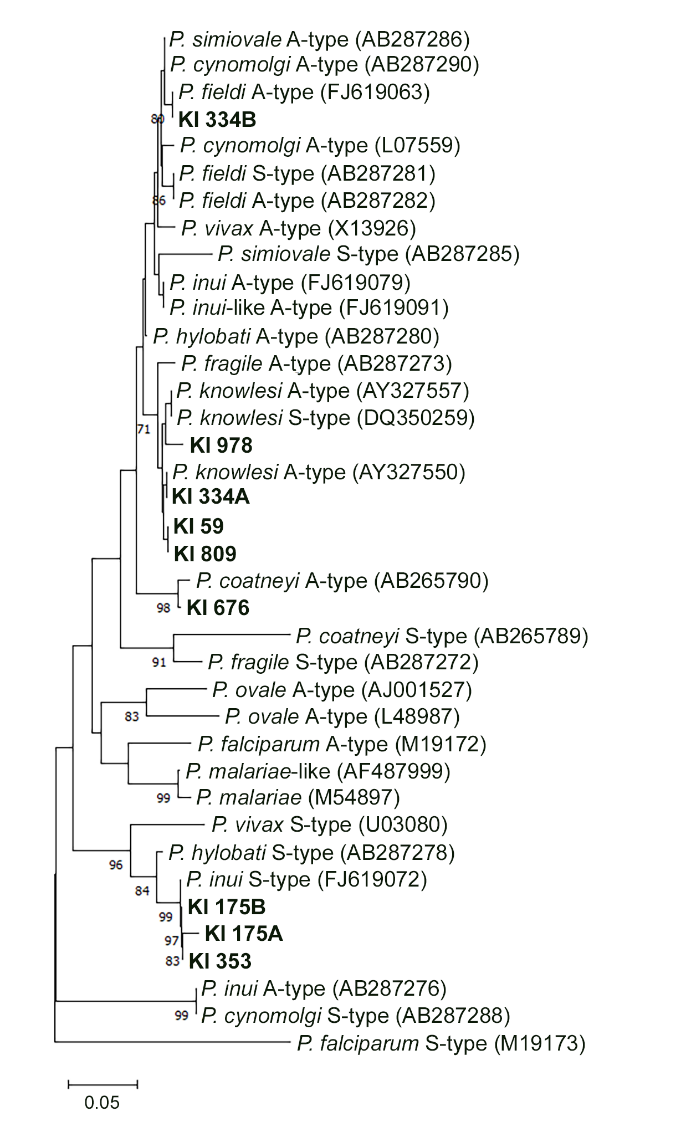 | 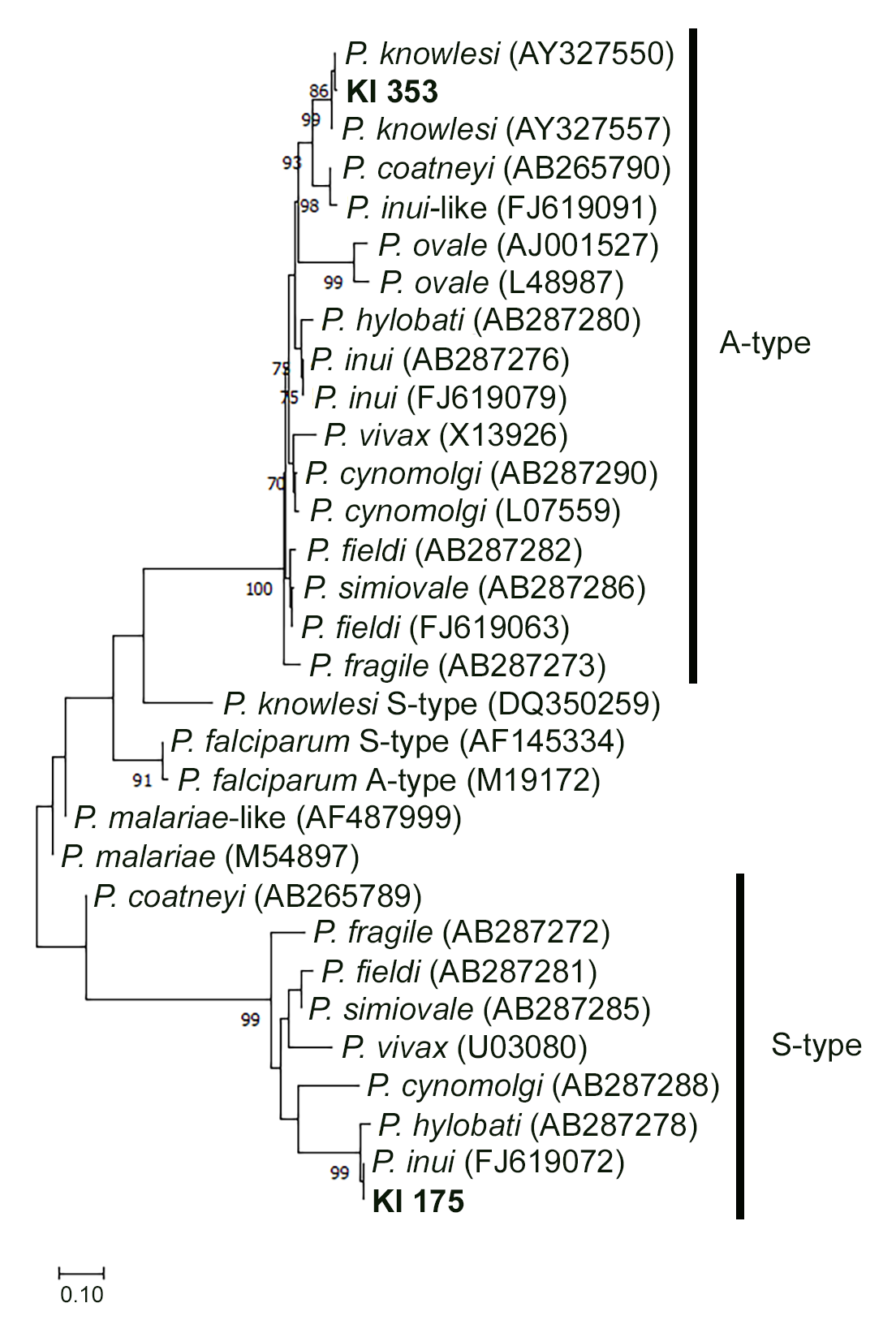 |
